# Supplementary material for: Comparative Analysis of Free-Circulating and Vesicle-Associated Plasma microRNAs of Healthy Controls and Early-Stage Lung Cancer Patients
Source: Pharmaceutics. 2022 Sep 23;14(10):2029. doi: 10.3390/pharmaceutics14102029 (PMC9610033; doi:10.3390/pharmaceutics14102029)
Supplement: Supplementary file 1 [file pharmaceutics-14-02029-s001.zip › Figure S1.pdf]

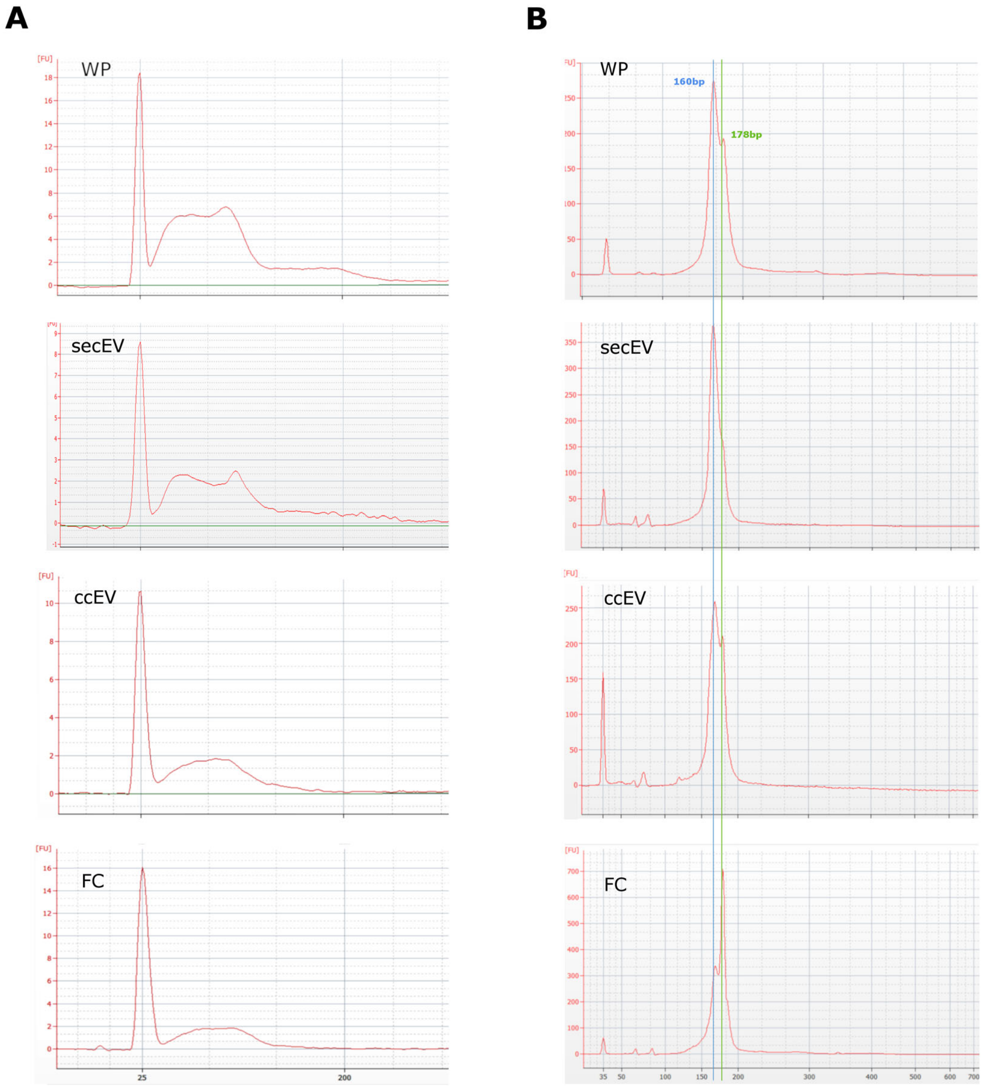

Figure S1. Bioanalyzer 2100 traces. (A) Size of RNA fragments detected in the four RNA extractions (RNA6000 pico kit). (B) dsDNA library size obtained using Qiaseq miRNA library kit starting from the four different types of RNA extractions (High Sensitivity DNA kit). The green bar at 178bp represents the expected library size of miRNA-positive DNA libraries.
